# Effect of virtual group EcoMeditation on psychological conditions and flow states

**DOI:** 10.3389/fpsyg.2022.907846

**Published:** 2022-11-15

**Authors:** Dawson Church, Peta Stapleton, Donna Gosatti, Tom O’Keefe

**Affiliations:** ^1^National Institute for Integrative Healthcare, Fulton, CA, United States; ^2^School of Psychology, Faculty of Society and Design, Bond University, Gold Coast, QLD, Australia

**Keywords:** meditation, anxiety, depression, PTSD, group therapy, virtual, EcoMeditation, emotional freedom techniques

## Abstract

**Background:**

A plethora of literature has delineated the therapeutic benefits of meditation practice on psychological functioning. A novel meditative practice, EcoMeditation, includes elements of four evidence-based techniques: The Quick Coherence Technique for regulating heart rate variability (HRV), Emotional Freedom Techniques (EFT), mindfulness, and neurofeedback.

**Objectives:**

Changes in psychological symptoms, including anxiety, depression, posttraumatic stress, pain, and happiness were measured following a one-day virtual EcoMeditation training workshop. The current study extended on previous literature by adding measures of transcendent experiences and flow states.

**Methods:**

Participants were drawn from a convenience sample of 151 participants (130 female, 21 male) aged between 26 to 71 years (*M* = 45.1, *SD* = 9.19) attending a one-day virtual EcoMeditation workshop. They were assessed pre-workshop, post-workshop, and at 3-months follow-up.

**Results:**

Post-workshop results (*N* = 111) indicated a significant reduction in anxiety (−42.3%, *p* < 0.001), depression (−37.5%, *p* < 0.001), posttraumatic stress (−13.0%, *p* < 0.001), and pain (−63.2%, *p* < 0.001) Likert mean scores when compared to pre-workshop. There was also a significant increase in happiness (+111.1%, *p* < 0.001), flow states (+17.4%, *p* < 0.001), and transcendent experiences (+18.5%, *p* < 0.001). At 3-months follow-up, a one-way repeated measures ANOVA (*N* = 72) found significant decreases in anxiety, depression, and pain symptoms between pre-test and post-test, as well between pre-test and follow-up. Flow, happiness, and transcendent experiences increased significantly between pre-test and post-test, as well as between pre-test and follow-up, with over 71% of participants experiencing clinically significant improvements. Significant reductions in posttraumatic stress and depression symptoms between pre-test and follow-up were also noted.

**Conclusion:**

EcoMeditation is associated with significant improvements in psychological conditions such as anxiety, depression, pain, and posttraumatic stress. EcoMeditation was also shown to enhance flow states and transcendent experiences. The benefits identified were similar to those found in the existing literature and provide support for the use of EcoMeditation as an effective stress reduction method that improves psychological symptoms and enhances transcendent states.

## Introduction

A plethora of meditation literature has delineated the therapeutic benefits of meditation practice on psychological functioning (McGee, 2008; Ospina et al., 2008; Sedlmeier et al., 2012). Meditation is a practice designed to increase mental awareness, clarity, and calmness using an array of techniques such as mindfulness, mantra recitation, breathwork, and movement (Ospina et al., 2008; Church, 2020). Empirical studies have found that mindfulness-based interventions assist with emotional regulation, self-care, and mood (Chambers et al., 2007; Jain et al., 2007; Shapiro et al., 2007; Robins et al., 2011; Sedlmeier et al., 2012; Ding et al., 2014). Significant reductions in anxiety, depression, psychological distress, and pain have been identified following meditative practices (Shapiro, 2009; Bohlmeijer et al., 2010).

In India’s Indus Valley approximately 5,000 BCE, archaeologists have revealed images of humans in meditative postures, with crossed legs, hands on knees, and eyes closed. However, it is likely that these practices originated much earlier, indicating that meditative experience has been valued by human beings for millennia (Puff, 2013). While the benefits of individual meditative practice has been well-supported in the literature, the psychological outcomes of group-based meditative practices has been less extensively explored in non-meditator samples (Fredrickson et al., 2008). It has been recommended that research expands on the social and relational aspects of meditation, with the assumption that the presence of others can enhance concentration, focus, and deepen the individual meditative experience (Cialdini and Goldstein, 2004; Vieten et al., 2018). Given the established cognitive impact of meditation, investigation of virtual and group-delivered models is warranted.

EcoMeditation is an emergent meditative practice derived from the Whole Energy Lifestyle (WEL) suite of evidence-based stress reduction and interpersonal relationship skills (Church, 2011). WEL was developed to combine evidence-based practices to move the field of energy healing above baseline (Church et al., 2020). WEL includes elements drawn from qigong, Gestalt therapy, yoga, and other evidence-based techniques (McCraty, 2005; Chiesa and Serretti, 2009; Jahnke et al., 2010; Feinstein, 2012; Church et al., 2014), which can be practiced supplementally to EcoMeditation (Church, 2011). In particular, EcoMeditation includes elements of four evidence-based techniques: The Quick Coherence Technique for regulating heart rate variability (HRV), Emotional Freedom Techniques (EFT), mindfulness, and neurofeedback (Davidson et al., 2003; Zotev et al., 2011; Church, 2013; McCraty and Zayas, 2014). The combined efficacy of the four empirical methodologies has only recently been tested (Church et al., 2020). Hence, the current study aimed to extend the findings of the existing literature to further establish the efficacy of EcoMeditation.

EcoMeditation instructs practitioners to imitate the breathing patterns and body postures of an experienced meditator. No prior training is necessary. A number of benefits have been identified with the practice. Pennington et al. (2019) identified advanced brainwave patterns using electroencephalogram (EEG), specifically increased gamma synchrony between the left and right hemispheres, during participants’ first EcoMeditation experience. In addition, Groesbeck et al. (2018) explored psychological and physiological markers during a two-day EcoMeditation workshop and identified several health benefits. The study found significant reductions in anxiety, depression, and pain, along with a decrease in physiological measures of stress (i.e., cortisol, resting heart rate). However, due to a modest sample size (*N* = 34) not all measures reached statistical significance.

A randomized controlled trial using fMRI compared EcoMeditation to mindful breathing (Church et al., 2022). Participants in the experimental and control group listened to 22-min audio tracks for 28 days. The EcoMeditation group showed posttest changes in functional connectivity in several brain regions while no alterations were found in the control group. Activity diminished in the part of the default mode network typically identified with self-referential thinking and decreased happiness, the mid-prefrontal cortex. However, activity increased in the brain region associated with prosocial emotions and compassion, the insula (Church et al., 2022). The brain changes are typical of those found in studies of experienced meditators, such as Tibetan monks with 10,000 h of practice (Goleman and Davidson, 2017). The novelty of this study was that similar patterns were identified in novices after four weeks of EcoMeditation practice.

EcoMeditation consists of ideologically neutral, evidence-based physiological relaxation cues (e.g., “relax your tongue on the floor of your mouth.”). It avoids language associated with philosophy, worldview, belief, religion, or spiritual practice (Pennington et al., 2019). Psychological benefits are often reported by novice practitioners after the first attempt, which increases long-term compliance of the practice. Since the development of the method by the first author in 2009, EcoMeditation has been made freely available. It can be downloaded online (www.EcoMeditation.com) which in turn makes it accessible to any aspiring meditator who wishes to experience it.

The distractions of “monkey mind” – the mind’s tendency to jump from subject to subject when not focused on a task - present a notoriously difficult challenge even for seasoned mediators (Goleman and Davidson, 2017). EcoMeditation circumvents this obstacle, as it does not require meditators to attenuate the stream of thoughts that move through daily human consciousness. Instead, the techniques are designed to provide the mind with an alternative focus. Quieting the mind is associated with a reduction of activity in the brain’s default mode network (Cheng et al., 2020). This is one of the brain regions that the aforementioned fMRI study found to be significantly downregulated (Church et al., 2022). Previous literature has found that the brain’s task-positive network automatically suppresses the activity of the default mode network when active (Cheng et al., 2020). The instructions for EcoMeditation are intended to take advantage of this phenomenon by keeping the task-positive network engaged using breathing exercises and guided imagery.

A previous study found robust psychological benefits from a one-day EcoMeditation workshop conducted using an in-person group format (Church et al., 2022). While the current study used the same one-day format, virtual delivery was implemented. To promote engagement, participants were instructed using a set of practices termed “active learning.” This included techniques such as repeating key concepts to a partner in a virtual breakout room, posting questions and comments in chat, and providing wellbeing scores after exercises. Research shows that classes using this model produce better student comprehension than conventional lectures (Deslauriers et al., 2019).

**Figure 1:**
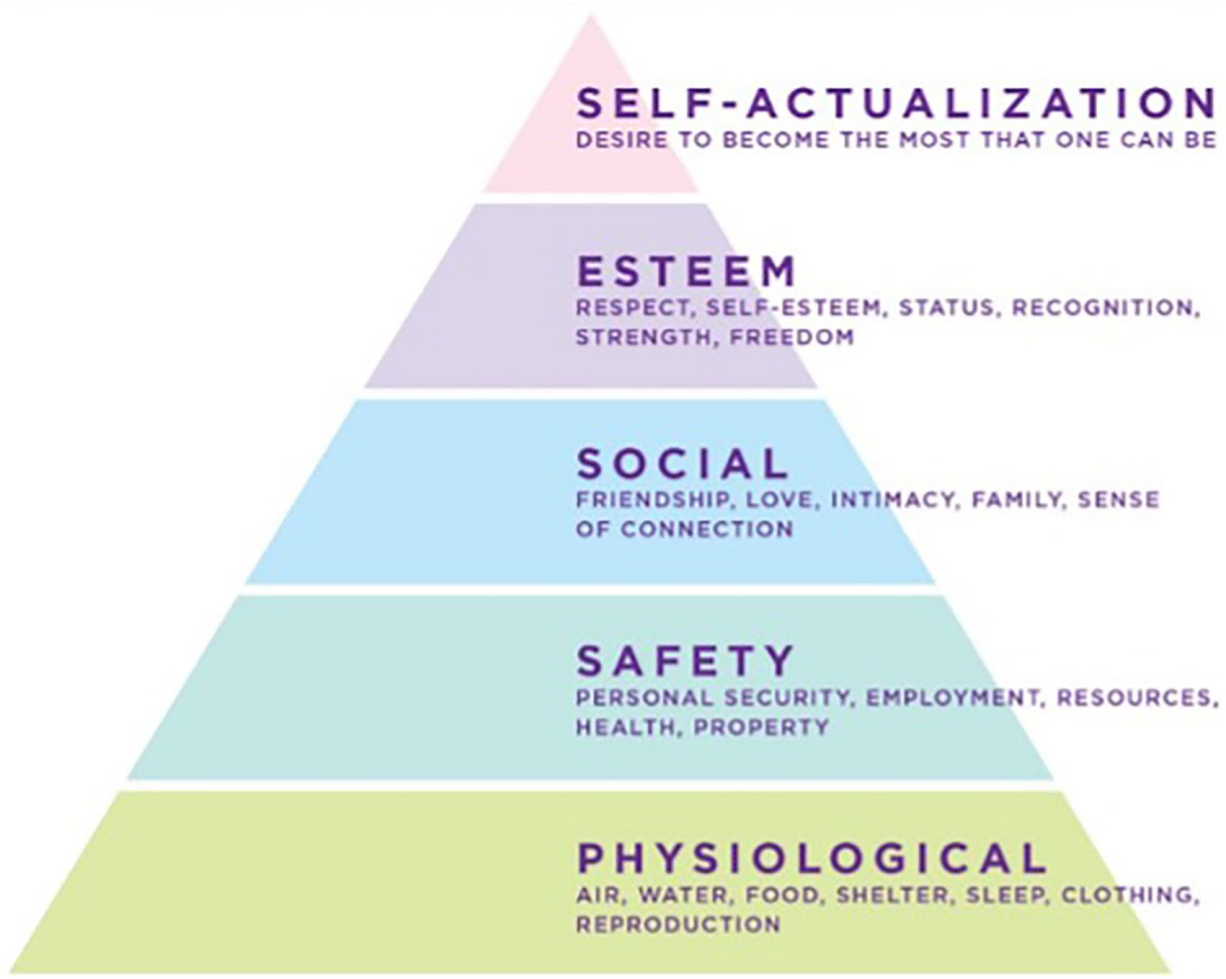
The traditional model of Maslow’s “Hierarchy of Needs” (Maslow, 1943).

Furthermore, this study extended the scope of previous research by collecting data on flow states and transcendent experiences, or what psychologist Abraham Maslow called “peak experiences” (Mathes, 1981). One of the iconic images of the human potential movement is Maslow’s “hierarchy of needs” model (see Figure 1; Maslow, 1943). Survival needs, such as air, food, and water are at the base of the pyramid. Once these needs are met, higher-order goals, such as social relationships and self-esteem can be pursued.

**Figure 2:**
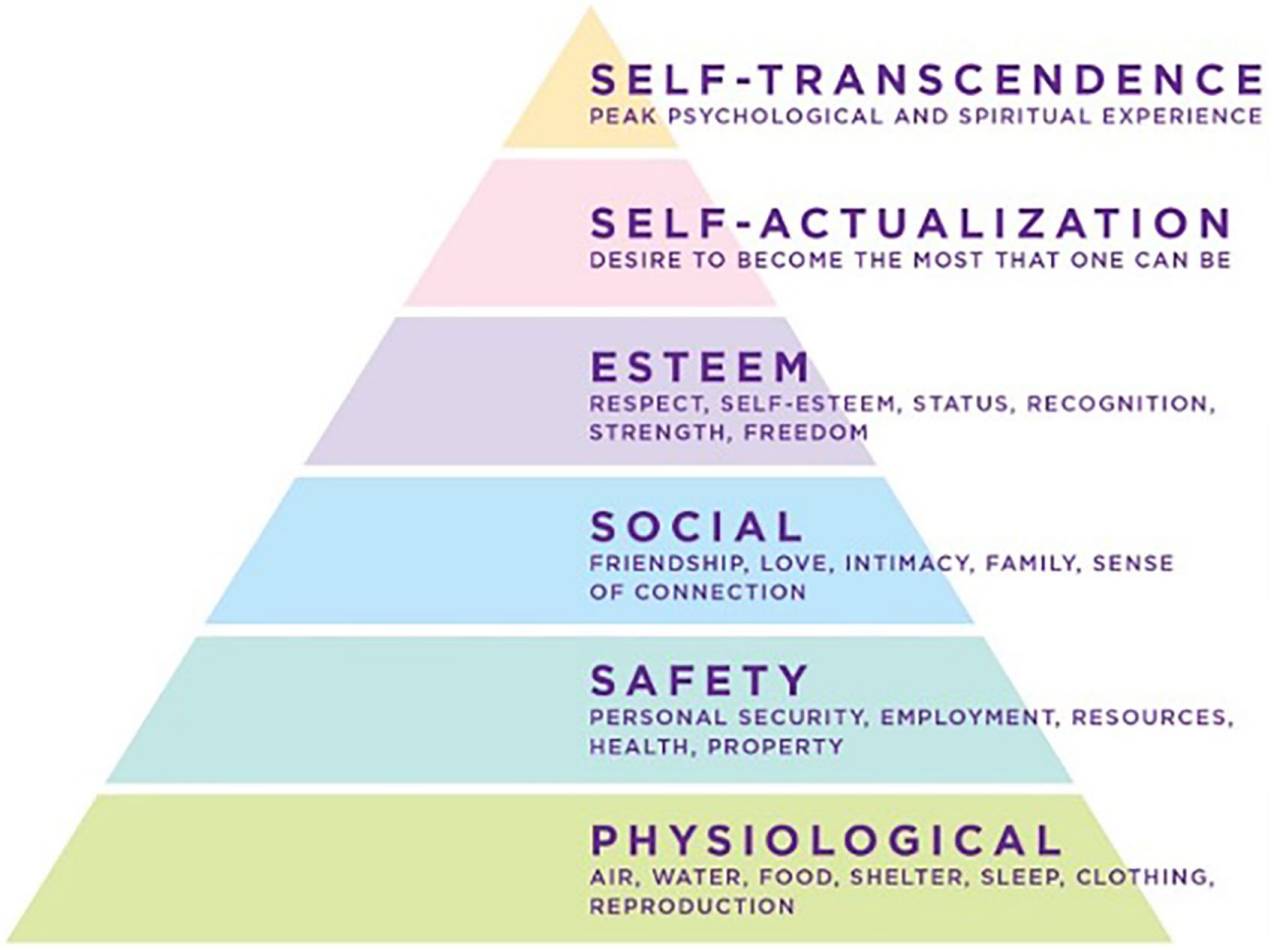
Maslow’s revised model of the “Hierarchy of Needs” (Koltko-Rivera, 2006).

Later, Maslow identified peak experiences beyond the self-actualization level that he had placed at the summit of the pyramid (see Figure 2; Church, 2022). Maslow termed his new capstone “self-transcendence.” The word “transcendence” is conventionally defined as an experience beyond or above the range of normal human experience in the material universe (Oxford English Dictionary, 2022). Self-transcendence is an experience of identity beyond the material self. It is described by meditation adepts as “no-self” and in the Sufi tradition as the “dissolution of the ego” (Vidich, 2015). This state has been a consistent phenomenological experience described by meditation adepts over the course of millennia (Newberg and Waldman, 2017).

Recent brain imaging advances have allowed investigators to progress beyond subjective self-report to objective measurement of the neural correlates of self-transcendent experiences. MRI studies of monks with over 10,000 h of meditation practice show a reduction in default mode network brain activity during meditation. Activity in the mid-prefrontal cortex, critical to constructing the sense of “self,” reduced greatly; the phenomenological experience of “no self” reported by these adepts is reflected in the attenuation of activity in these specific regions of their brains (Newberg and Waldman, 2017).

The second brain region notable in self-transcendent experiences is the insula. When long-term meditators focus on compassion, activity in the insula increases. The insula is highly active during the experience of pro-social and positive emotions including compassion, gratitude, and love. Here again, the activity of the brain matches the phenomenological self-report of meditation practitioners. The previous fMRI study of EcoMeditation identified similar patterns in the insula and mid-prefrontal cortices of participants (Church et al., 2022).

Maslow used the term “peak experiences” to describe the phenomenology of self-actualized and self-transcendent states. This was later amplified by Nakamura and Csikszentmihalyi (2014) and modelled as “flow.” Flow is an experience “in which an individual is completely immersed in an activity without reflective self-consciousness but with a deep sense of control” (Engeser et al., 2021). Flow research was one of the primary influences motivating the emerging field of positive psychology (Engeser et al., 2021). Csikszentmihalyi characterised flow as the “optimal experience” in the sense that it is defined as “a psychological state in which the person feels simultaneously cognitively efficient, motivated, and happy” (Moneta and Csikszentmihalyi, 1996).

While the relief of dysphoric states such as anxiety and depression have undoubted psychological utility, adding a measure of flow to a study addresses the capstone of Maslow’s revised pyramid, the dimension of self-transcendence with its peak in psychological and spiritual experiences. Such measures allow investigators to determine whether an intervention both ameliorates emotionally distressing states and promotes peak states. For these reasons, the investigators in the current study built upon the existing scientific literature by adding measures of flow and transcendent experiences.

### Aims and hypotheses

Thus, the current study aimed to extend Church et al. (2022)’s paper to examine the psychological effects of a one-day EcoMeditation intervention delivered in virtual group format. This study also explored changes in flow states and transcendent experiences to identify the co-occurrence of Maslow’s “peak experiences” as described in the relevant literature.

## Materials and methods

### Participants and procedure

The study was reviewed by the Ethics Committee of the National Institute for Integrative Healthcare (NIIH20181101) and found to present minimal risk to participants. A convenience sample of 151 participants engaged in a one-day virtual EcoMeditation training workshop. The majority of the sample identified as female (*n* = 130, 85%) and participants ranged between 26 and 71 years old (*M* = 45.1, *SD* = 9.19). More than 50% had completed at least a high school level education. All provided consent by ticking a box on a virtual consent form prior to completing assessments. Following that, questionnaires were completed. Participants were required to provide consent and instructed to complete assessments pre-workshop, post-workshop, and at 3-months follow-up. Table 1 contains the demographic data for the sample.

**Table 1:** Frequencies and percentages of the virtual workshop demographics (*N* = 151).

Variable	Category	*n* (%)
Age (years)	25–34	21 (13.9)
	35–44	51 (33.8)
	45–54	56 (37.1)
	55–64	21 (13.9)
Level of Education	65+	2 (1.32)
	Elementary	27 (17.9)
	High School	61 (40.4)
	College	56 (37.1)
	Graduate	7 (4.6)

*n* = Total number of participants in each group, % = Percentage of participants in each group.

### Measures

The workshop registration process included the collection of demographic information such as age (years), gender, highest level of education (elementary, high school, college, or graduate) and contact details. Eight items from valid and reliable instruments assessed psychological symptoms of anxiety, depression, posttraumatic stress, general happiness, and pain as described below.

#### Anxiety

Two items from the Generalised Anxiety Disorder-7 inventory (GAD-7; Spitzer et al., 2006) measured participants’ anxiety symptoms over the prior 2 weeks. Items were scored using a 4-point Likert scale, ranging from 0 (*not at all*) to 3 (*nearly every day*). A total anxiety score was derived from the summation of the two items (0–6), with higher scores indicative of greater symptoms. The GAD-7 is a valid and reliable measure used to screen for GAD in clinical research, with a score of 3 or greater indicating the likelihood of anxiety disorders (Donker et al., 2011). The PHQ-4 has demonstrated that the two anxiety items of GAD-2 indicated 84% of the total variance was explained by the first two factors (Kroenke et al., 2009).

#### Depression

Two items from the Patient Health Questionnaire-2 (PHQ-2; Kroenke et al., 2003) were included to assess symptoms of depression over the prior 2 weeks. Items were scored using the 4-point Likert scale, ranging from 0 (*not at all*) to 3 (*nearly every day*). A total depression score was derived from the summation of the two items (0–6), with higher scores indicative of greater symptoms. The measure has demonstrated good sensitivity (79%) and specificity (86%) for screening, with a score of 3 or greater indicating the likelihood of depressive disorders (Löwe et al., 2005). Internal reliability (Cronbachs α) was deemed good (>0.80) for PHQ-2 (Kroenke et al., 2009).

#### Posttraumatic stress

The two-item PTSD Checklist (PCL-2; Lang et al., 2012) was used to assess symptoms of PTSD in the prior month. Items were scored using a 5-point Likert scale, ranging from 1 (*not at all*) to 5 (*extremely*). A total PTSD score was derived from the summation of the two items, with scores ranging from 2 to 10. Higher scores indicated potential clinical levels of psychological distress, with a score of 4 indicating a probable PTSD diagnosis. The PCL-2 has high sensitivity and provides a reliable indicator of significant clinical change (Lang et al., 2012).

#### Happiness

The 1-point Happiness Scale was used to assess participants’ general happiness. Scores range from 0 (*not at all*) to 10 (*very*). Though brief, it has been found to correlate with extensive happiness instruments (Abdel-Khalek, 2006).

#### Pain

The 1-point Numeric Pain Rating Scale was used to measure the intensity of the current, best, and worst pain levels experienced in the past 24 h, ranging from 0 (*not at all*) to 10 (*worst pain imaginable*; McCaffery and Beebe, 1989).

#### Flow states and transcendent experiences

Ten items from the Flow Short Scale measure components of the flow experience to assess participants’ most recent level of flow (Rheinberg et al., 2008). Items are scored using a 7-point Likert scale, ranging from 1 (*not at all*) to 7 (*very much*). A total flow state score was obtained from the summation of the 10-items (10–70), with higher scores indicating an increased flow state. The 5-item Universal Experiences Scale (Church and Stapleton, 2022) is based on the 5 characteristics of transcendent experiences reported by Newberg in a sample of 2,000 online surveys (Newberg and Waldman, 2017). It is designed to be a short instrument, in contrast to longer assessments such as the Mystical Experiences Questionnaire (MEQ) which has 30 items (Hood, 1975). Items are scored using an 11-point Likert scale, where scores range from 0 (*completely untrue*) to 10 *(completely true*). A total score was obtained from the summation of the 5-items (0–50), with higher scores indicating an increased level of transcendent experiences.

### EcoMeditation training and practice

EcoMeditation consists of stress-reduction skills, specifically mindfulness, heart coherence, EFT, and neurofeedback. Participants attended a full-day virtual workshop and completed assessments before and immediately after the session. In the workshop, participants were provided with information regarding research into meditation followed by the practice of EcoMeditation and group feedback. The workshop consisted of four 90-min modules, with breaks in between. Each module reviewed a component of meditation research, such as the physiology of stress, the brain regions active in meditation, and the brainwave profiles of various moods.

Each presentation was followed by a guided EcoMeditation of about 30 min duration. Participants closed their eyes and performed each of the evidence-based techniques referenced above. These included EFT, the Quick Coherence Technique, and mindful interoceptive awareness. Emotionally neutral music was played virtually in the background. The first author, the developer of EcoMeditation, administered the six-hour virtual training workshop alongside other trained practitioners, and provided EcoMeditation instruction and guidance to participants in their home environment. To close, participants were provided with handouts and instructions to practice EcoMeditation at home following the workshop.

## Results

The data was analysed using Statistical Package for Social Sciences Version 28 (SPSS; IBM, Armonk, NY, 2014). An alpha level of.05 was used for all statistical analyses unless otherwise specified.

### Psychological markers

**Figure 3:**
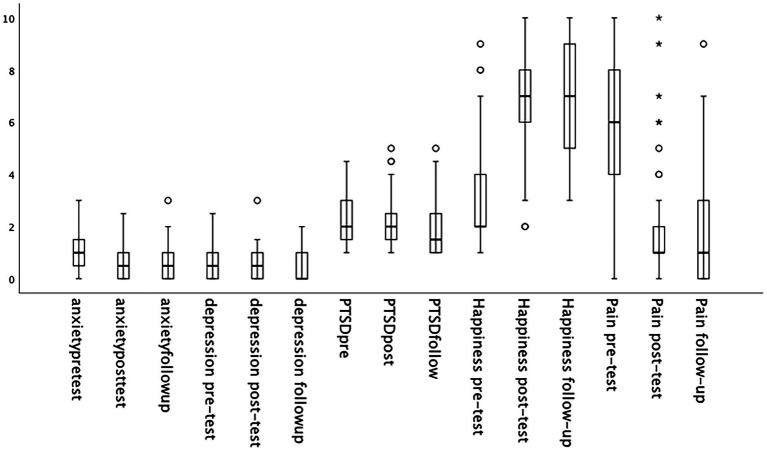
The distribution of data and outliers present across psychological markers across time (*N* = 151).

*A priori* analysis using the program G*Power 3.1 indicated that the sample (*N* = 151) was sufficient to ensure adequate power (80% statistical power, *f*2 = 0.25). Prior to main analyses, the data was screened. While the visual inspection of box plots indicated several univariate outliers, the values revealed to be genuinely unusual scores were retained for further analyses (see Figure 3). Several participants reported higher pain levels posttest. The Shapiro–Wilk test results indicated violations to the assumption of normality, and therefore pretest and posttest scores were compared using Wilcoxon Signed Rank Tests for paired samples.

The overall results indicated a significant reduction in anxiety, depression, PTSD, and pain scores from pretest to posttest. Following EcoMeditation training, there was also a significant increase in happiness scores (see Table 2).

**Table 2:** Measures outcomes pre- and post-EcoMeditation training (*N* = 111).

Scale	Pre-test Mean ± SD	Post-test Mean ± SD	Change in mean	*t statistic*	*p* value
Anxiety	1.04 ± 0.85	0.60 ± 0.58	−0.44	6.54	< 0.001
Depression	0.72 ± 0.74	0.45 ± 0.59	−0.27	4.56	<0.001
PTSD	2.30 ± 0.96	2.0 ± 0.92	−0.30	4.13	<0.001
Happiness	3.34 ± 2.31	7.05 ± 1.90	+3.71	−13.51	<0.001
Pain	5.57 ± 2.76	2.06 ± 2.18	−3.52	10.93	<0.001

In addition, a series of one-way repeated measures ANOVAs were performed to determine whether there were significant differences in anxiety, depression, PTSD, happiness, and pain across time. The sample consisted of 57 participants who had completed the assessments at all three timepoints including pre-workshop, post-workshop, and 3-months follow-up. *A priori* power analysis indicated that the sample (*N* = 57) was sufficient to ensure adequate power (80% statistical power, *f*2 = 0.25). The Shapiro–Wilk test indicated a violation to the assumption of normality; However, a slight skew has minimal influence on ANOVA analyses due to its robust nature. Assumptions of homogeneity of variance were met. A Hunynh-Feldt correction was applied where Mauchly’s test of sphericity was violated.

### Anxiety

A significant difference between anxiety scores was found between the pretest (*M* = 1.11, *SD* = 0.83), posttest (*M* = 0.68, *SD* = 0.58), and follow-up timepoints (*M* = 0.68, *SD* = 0.64), *F*(1.64, 92) = 21.33, *p* < 0.001, partial η^2^ = 0.28. *Post hoc* analysis with a Bonferroni adjustment indicated a significant decrease in anxiety scores from pretest to posttest (*M* = 0.44, 95% CI [0.22, 0.66], *p* < 0.001) and pretest to follow-up (*M* = 0.44, 95% CI [0.24, 0.64], *p* < 0.001). There were no significant changes in anxiety between posttest and follow-up (*M* = 0.95% CI [−0.14, 0.14], *p* = 1.00), indicating that participants maintained the gains they had made during the workshop over time.

### Depression

There were a significant difference in depression scores from pretest (*M* = 0.65, *SD* = 0.63), posttest (*M* = 0.45, *SD* = 0.59), and follow-up (*M* = 0.40, *SD* = 0.53), *F*(1.73, 106.92) = 7.49, *p* = 0.002, partial η^2^ = 0.11. *Post hoc* analysis with Bonferroni adjustment indicated significant decrease in depression scores from pretest to posttest (*M* = −0.20, 95% CI [0.01, 0.39], *p* = 0.038) and pretest to follow-up (*M* = −0.25, 95% CI [0.07, 0.44], *p* = 0.004). There were no significant changes in depression scores from posttest to follow up (*M* = 0.06, 95% CI [−0.07, 0.18], *p* = 0.868), again indicating durable maintenance of participant gains.

### Posttraumatic stress

Mean PTSD scores significantly decreased over time, *F*(2, 112) = 7.21, *p* = 0.001, partial η^2^ = 0.11, from pretest (*M* = 2.30, *SD* = 0.93), posttest (*M* = 2.06, *SD* = 0.95), and follow-up (*M* = 1.92, *SD* = 0.94). *Post hoc* analysis with Bonferroni adjustment revealed a significant decrease in PTSD mean scores from pretest to follow-up, (*M* = −0.38, 95% CI [0.12, 0.64], *p* = 0.002), but not from pretest to posttest, (*M* = −0.24, 95% CI [−0.02, 0.49], *p* = 0.080) and posttest to follow-up (*M* = −0.14, 95% CI [−0.08, 0.37], *p* = 0.385).

### Happiness

There were significant differences in happiness means scores from pretest (*M* = 3.29, *SD* = 2.14), posttest (*M* = 7.00, *SD* = 1.91), and follow-up (*M* = 7.17, *SD* = 1.94), *F*(2, 116) = 84.26, *p* < 0.001, partial η^2^ = 0.59. *Post hoc* analysis with Bonferroni adjustment revealed a significant increase in happiness from pretest to posttest (*M* = −3.71, 95% CI [−4.70, −2.73], *p* < 0.001) and pretest to follow-up (*M* = −3.88, 95% CI [−4.83, −2.93], *p* = < 0.001). There were no significant changes in happiness scores from posttest to follow-up (*M* = −0.17, 95% CI [−0.64, 0.30], *p* = 1.00), indicating a maintenance of participant gains.

### Pain

Mean pain scores significantly decreased from pretest (*M* = 5.69, *SD* = 2.69), posttest (*M* = 1.97, *SD* = 2.27), and follow-up (*M* = 1.97, *SD* = 2.25), *F*(2, 116) = 54.59, *p* < 0.001, partial η^2^ = 0.49. *Post hoc* analysis with Bonferroni adjustment revealed a significant decrease in pain scores from pretest to posttest (*M* = 3.73, 95% CI [2.58, 4.88], *p* < 0.001) and pretest to follow-up (*M* = 3.73, 95% CI [2.52, 4.93], *p* < 0.001). There were no significant changes in pain scores from posttest to follow-up (*M* = 0, 95% CI [−0.58, 0.58], *p* = 1.00), again indicating the durability of the results obtained by participants in the workshop.

## Flow states and transcendent experiences

**Figure 4:**
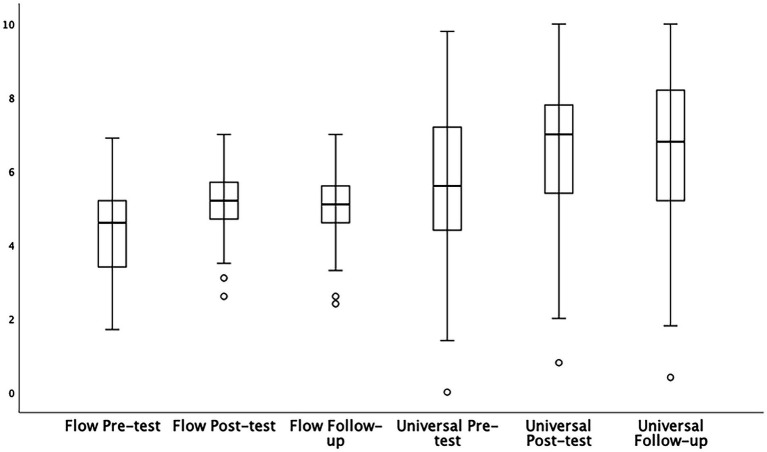
The distribution and outliers of flow states and transcendent experiences over time (*N* = 151).

Prior to main analyses, the distribution of scores were roughly symmetrical and no extreme univariate outliers were detected (see Figure 4). The Shapiro–Wilk test indicated a violation to normality, therefore, Wilcoxon Signed Rank Tests for paired samples were used.

The results indicated a significant increase in flow state scores from pretest (*M* = 4.32, *SD* = 1.06) to posttest (*M* = 5.07, *SD* = 0.91), *t* = −8.18, *p* < 0.001. Also found was a significant increase in experiences typical of transcendence from pretest (*M* = 5.61, *SD* = 2.16) to posttest (*M* = 6.65, *SD* = 1.94), *t* = −6.46, *p* < 0.001.

In addition, one-way repeated measures ANOVAs were performed to determine whether there were significant differences in flow states and transcendent experiences across time. Although a violation to normality was detected by the Shapiro–Wilk test, no transformations were conducted due to ANOVAs robust nature. Assumptions of homogeneity of variance were met. If Mauchly’s test of sphericity was not met, a Hunynh-Feldt correction was applied.

### Flow states

A significant difference in flow state scores was found between pretest (*M* = 4.37, *SD* = 1.10), posttest (*M* = 5.12, *SD* = 0.90), and follow-up (*M* = 4.99, *SD* = 0.94), *F*(2, 116) = 25.35, *p* < 0.001, partial η^2^ = 0.30. *Post hoc* analysis with a Bonferroni adjustment indicated a significant increase in flow state from pretest to posttest (*M* = −0.76, 95% CI [−1.06, −0.45], *p* < 0.001) and pretest to follow-up (*M* = −0.63, 95% CI [−0.91, −0.34], *p* < 0.001). There were no significant changes in flow state between posttest and follow-up (*M* = 0.13, 95% CI [−0.12, 0.38], *p* = 0.623), indicating the durability of participant gains over time.

### Transcendent experiences

A significant difference in transcendent experience scores was found between pretest (*M* = 5.66, *SD* = 2.27), posttest (*M* = 6.66, *SD* = 1.98), and follow-up (*M* = 6.61, *SD* = 2.15), *F*(2, 116) = 13.89, *p* < 0.001, partial η^2^ = 0.19. *Post hoc* analysis with a Bonferroni adjustment indicated a significant increase in transcendent experience from pretest to posttest (*M* = −0.99, 95% CI [−1.57, −0.42], *p* < 0.001), and pretest to follow-up (*M* = −0.95, 95% CI [−1.38, −0.52], *p* < 0.001). There were no significant changes in transcendence between posttest and follow-up (*M* = 0.04, 95% CI [−0.51, 0.60], *p* = 1.00), again indicating that participant gains were durable.

## Clinical significance of happiness, transcendent experiences, and flow state scores

To report the size of the effect of treatment in standardized terms, Cohen’s conventions for modest, moderate, and large differences (respectively, *d* = 0.2, *d* = 0.5, *d* = 0.8+) were employed (Cohen, 1988). If the mean difference in pre-post scores changed by at least 2 Likert points, a clinically significant improvement was identified (Ranganathan et al., 2015; Martin et al., 2021). For happiness, 77.5% (*N* = 86) of participants reported a significant improvement from pre-test (*M* = 3.34, *SD* = 2.31) to posttest (*M* = 7.05, *SD* = 1.90) which produced a large treatment effect size (*M* = +3.70, *d* = 1.28). For flow states and transcendent experiences, 78.4% (*N* = 87) and 71.2% (*N* = 79) of participants reported significant change, respectively. A moderate effect size was identified for flow state mean scores from pretest (*M* = 4.32, *SD* = 1.06) to posttest (*M* = 5.07, *SD* = 0.91; *M* = +0.75, *d* = 0.78) and transcendent experiences from pretest (*M* = 5.61, *SD* = 2.16) to posttest (*M* = 6.65, *SD* = 1.94; *M* = +1.04, *d* = 0.61).

## Discussion

The current study measured the psychological benefits, flow states, and transcendent experiences associated with EcoMeditation, a method combining four evidence-based stress-reduction techniques. After a one-day EcoMeditation workshop, results indicated that participants (*N* = 151) experienced a significant reduction in anxiety, depression, PTSD, and pain, along with an increase in happiness, flow states and transcendent experiences. At 3-months follow-up, one-way repeated measures ANOVAs (*N* = 57) indicated a significant decrease in anxiety, depression, and pain between pretest and posttest, as well as pretest and follow-up. The results also revealed a significant increase in flow states, happiness, and transcendent experiences between pretest and posttest, as well as pretest and follow-up. Decreased PTSD symptoms were evident over the 3-month period between pretest and follow-up.

The current study provided a differentiated examination of EcoMeditation by using a virtual group delivery format. It extended previous literature by including measures of flow states and transcendent experiences. Overall, the empirical findings support those of previous studies demonstrating that EcoMeditation yields improvements in psychological outcome measures of anxiety, depression, pain, happiness, and PTSD (Groesbeck et al., 2018; Pennington et al., 2019; Church et al., 2020, 2022). EcoMeditation was also associated with the benefits of increased flow and transcendent experiences, with over 71% of participants experiencing clinically significant improvements after the workshop. This indicates that EcoMeditation does more than ameliorate dysphoric psychological symptoms; it catalyses peak experiences.

There are several possible reasons why the virtual EcoMeditation workshop yielded greater efficacy for reducing PTSD and depression over the 3 months of the follow-up period. Participants were encouraged to practice EcoMeditation after the workshop, and were provided with resources to promote daily practice. At the end of the workshop, virtually all participants made a commitment to a consistent daily practice. Regular meditation might have enhanced therapeutic outcomes. In addition, participants practicing in their familiar environments might have felt more comfortable than in a public setting.

The study had several limitations. Vieten et al. (2018) proposed that the psychological improvements found in group meditative experiences may be attributed to a supportive group dynamic, demand characteristics, or the stress-reducing effects of meditation rather than the practice itself. In addition, investigator allegiance might have contributed to the results, since the first author, the developer of EcoMeditation, administered the intervention. The absence of a control group meant that the contribution of the non-specific effects of any therapeutic intervention was likely to have played a part in the results obtained. EcoMeditation was not compared to a known efficacious therapy or a control group during the study. Therefore, there was no measure obtained on how the population may have responded to other forms of treatment or no treatment. EcoMeditation combines multiple techniques, and therefore, the contribution of each technique to its therapeutic effects is unknown.

In addition, the sample only consisted of self-selected participants with presumed high levels of motivation, which limits the generalisability of the study’s findings to a heterogenous population. The study also did not control for participants’ previous experience using EcoMeditation and other forms of meditation. Further, the sample size at follow-up was approximately one third of the size of the initial sample due to participant attrition. It is possible that non-responders did not experience any psychological benefits. Mitigating this limitation is the finding that high participant attrition rates are typical of online studies and that non-response rates (<85%) do not tend to bias reported outcomes (Couper et al., 2007; Church and Brooks, 2010; Tabachnick and Fidell, 2014). A further limitation is the large gender disparity between participants. This sample, in which 85% were female, might have skewed the results. Lastly, the frequency and duration of participant use of EcoMeditation between the workshop and the follow-up point was not measured. The results noted in the follow-up might have been due to participant use of EcoMeditation between these two points, rather than the effects of the workshop itself.

Despite these limitations, the results of the study are noteworthy. These findings support the use of virtual EcoMeditation workshops in clinical settings, such as group therapy clinics, hospitals, outpatient support groups, employee meetings, training centers, veterans’ organisations, drug rehabilitation centers, prisons, and other settings that promote stress management. EcoMeditation may be of benefit to outpatient services, as clinical experience has shown that simple and efficient techniques are more likely to be practiced than complicated protocols.

The results of this study suggest recommendations for future research. Dismantling and component studies can determine the contribution made by each modality of EcoMeditation. The method should be examined in randomly selected samples and compared to other known efficacious methods such as mindfulness-based stress reduction (MBSR), EFT, and cognitive behaviour therapy (CBT). Additional studies using randomisation and active control groups will illuminate its effectiveness when measured against these established therapies. Further exploration using larger samples and the assessment of previous meditation experience will provide greater insight into any effects unique to EcoMeditation.

The results obtained at 3-months follow-up reveal that most participant gains were durable. Measures of whether participants practiced EcoMeditation and how frequently, will provide insight into this question. Delivery by instructors other than the developer will illuminate whether EcoMeditation is as effective when offered by others. Objective data independent of participants’ self-reports should also be obtained, extending the findings of the one existing study that used physiological markers (Groesbeck et al., 2018). As psychological stress is concomitant with physiological markers, assessments such as cortisol, immunoglobulins, C-reactive protein, gene expression, microRNAs and interleukins will further illuminate the physiological dimensions of change (Thrall et al., 2007; Ruwaard et al., 2013). In the fMRI study summarised above (Church et al., 2022), participants listened to a 22-min recorded audio tape rather than live training; nonetheless significant improvements in psychological and neurological function were identified. Live training in EcoMeditation should be contrasted with recorded EcoMeditation tracks, and in-person with virtual workshops.

## Conclusion

The results of this study are consistent with those of other studies (Groesbeck et al., 2018; Pennington et al., 2019; Church et al., 2020, 2022). EcoMeditation produces robust improvements in a range of psychological symptoms, including anxiety, depression, pain and PTSD. This study extended those investigations, and found that transcendent experiences and flow states were enhanced by EcoMeditation. Participant gains persisted 3-months after the EcoMeditation workshop. The four evidence-based techniques combined in EcoMeditation are associated with durable psychological benefits. Future research using randomised controlled trials is needed to establish whether the psychological gains identified in extant EcoMeditation studies can be replicated using more rigorous experimental designs, including the use of control and comparison studies.

## Data availability statement

The original contributions presented in the study are included in the article/Supplementary material, further inquiries can be directed to the corresponding author.

## Ethics statement

The studies involving human participants were reviewed and approved by National Institute for Integrative Healthcare Ethics Committee (NIIH20181101). The patients/participants provided their written informed consent to participate in this study.

## Author contributions

DC and PS designed the study, established scales, and recruited participants. DC facilitated the EcoMeditation workshops and collected participant data. DG performed statistical analyses and drafted the manuscript. TO’K assisted with forming final draft of manuscript. All authors contributed to the article and approved the submitted version.

## Funding

The authors disclosed receipt of the following financial support for the research, authorship, and/or publication of this article; Donations made to the National Institute for Integrative Healthcare (NIIH.org) funded the data gathering, analysis, and presentation of the results.

## Conflict of interest

The authors declare the following potential conflicts of interest with respect to the research, authorship, and/or publication of this article: DC receives income from EcoMeditation publications and presentations. DC shall declare he is the NIIH’s president/CEO, and Topic Editor for the Research Topic where this manuscript was submitted: The Future of Psychology: Approaches to Enhance Therapeutic Outcomes. Stapleton shall declare she is a NIIH Board member and Topic Editor for the Research Topic where this manuscript was PS: The Future of Psychology: Approaches to Enhance Therapeutic Outcomes.

The remaining authors declare that the research was conducted in the absence of any commercial or financial relationships that could be construed as a potential conflict of interest.

## Publisher’s note

All claims expressed in this article are solely those of the authors and do not necessarily represent those of their affiliated organizations, or those of the publisher, the editors and the reviewers. Any product that may be evaluated in this article, or claim that may be made by its manufacturer, is not guaranteed or endorsed by the publisher.
